# Antiphospholipid‐Related Chorea: Two Case Reports and Role of Metabolic Imaging

**DOI:** 10.1002/mdc3.13432

**Published:** 2022-03-28

**Authors:** Lisa Lerjefors, Silvia Andretta, Giulia Bonato, Michele Mainardi, Miryam Carecchio, Angelo Antonini

**Affiliations:** ^1^ Parkinson and Movement Disorders Unit, Centre for Rare Neurological Diseases (ERN‐RND), Department of Neuroscience University of Padua Padua Italy

**Keywords:** antiphospholipid, chorea, PET, MRI

## Abstract

**Background:**

Antiphospholipid syndrome (APS) is a complex acquired autoimmune disease with a wide clinical spectrum. Chorea is a rare neurological manifestation of APS.

**Cases:**

We report two elderly patients with APS‐related chorea in whom functional imaging (18F‐FDG positron emission tomography, FDG‐PET) supported the diagnosis and compare our findings with existing literature.

**Literature Review:**

Among 142 clinical cases of antiphospholipid‐related chorea found in literature, only 10 had undergone brain metabolic imaging. Striatal hypermetabolism was evident in all cases (6) that underwent FDG‐PET cerebral imaging. Cerebral perfusion single photon emission computed tomography (SPECT) was normal in two cases, while the other two presented with basal ganglia hypoperfusion.

**Conclusions:**

Brain FDG‐PET usually shows striatal hypometabolism in neurodegenerative types of chorea as opposed to striatal hypermetabolism observed in most cases of chorea from reversible etiologies, such as APS‐related chorea. When a patient's clinical presentation is not clearly suggestive of either a neurodegenerative or autoimmune chorea, and first‐line investigations are normal, FDG‐PET may help in the differential diagnosis, especially in the presence of striatal hypermetabolism. SPECT data are less numerous and show either normal scans or basal ganglia hypoperfusion.

## Case Series

Chorea is a hyperkinetic movement disorder caused by a large number of conditions, including inherited/degenerative, autoimmune, metabolic, and structural disorders as well as pharmacological treatments.^1^

Antiphospholipid syndrome (APS) is a complex acquired autoimmune disease, which can primarily lead to vascular thrombosis or pregnancy morbidity. Central nervous system manifestations of APS include cerebrovascular accidents, venous sinuses thrombosis, cognitive impairment and dementia, psychosis, seizures, movement disorders, headache, demyelinating disease, transverse myelitis and ischemic optic neuropathy.^2^ According to the results of the Euro‐Phospholipid study, that analyzed a cohort of 1000 APS patients, chorea had a prevalence of 1.3% among affected subjects.^3^ According to the Revised Sapporo Criteria for APS,^4^ positive test results for lupus anticoagulant (LAC), anticardiolipin antibodies (aCL) and/or anti‐β2‐glycoprotein I antibodies (β2‐GPI) are required for the diagnosis of APS, with laboratory confirmation after 12 weeks in the presence of consistent clinical and radiological findings. Cerebral magnetic resonance imaging (MRI) in APS‐related chorea is usually unremarkable; only rarely basal ganglia ischemic lesions are detected.^5^

We here report two elderly patients with APS‐related chorea in whom metabolic imaging (18F‐fluorodeoxyglucose positron emission tomography, FDG‐PET) supported the diagnosis and compare our findings with existing literature.

### Case 1

A 66‐year‐old man came to our attention because of generalized choreatic movements involving primarily the oromandibular region and the limbs with mild prevalence on the right side, without other neurological signs or cognitive complaints (Video 1). Onset was described as subacute. He had no relevant family history, was not on medication and had undergone surgery for an aortic biological prosthetic valve. Laboratory investigations showed no abnormality of blood cell count, coagulation, renal and hepatic function, copper metabolism, anti‐neuronal antibodies, antinuclear antibodies, peripheral blood smear, LAC and β2‐GPI antibodies. Anticardiolipin antibodies were at the upper normal limit. Genetic testing for Huntington's disease was negative. Brain MRI was unremarkable. Neuropsychological testing confirmed the absence of cognitive impairment.

**Video 1:** Patient 1 Neurological Examination. Video showing generalized choreatic movements involving primarily the oromandibular region and the limbs, with mild prevalence on the right side.

**FIG. 1:**
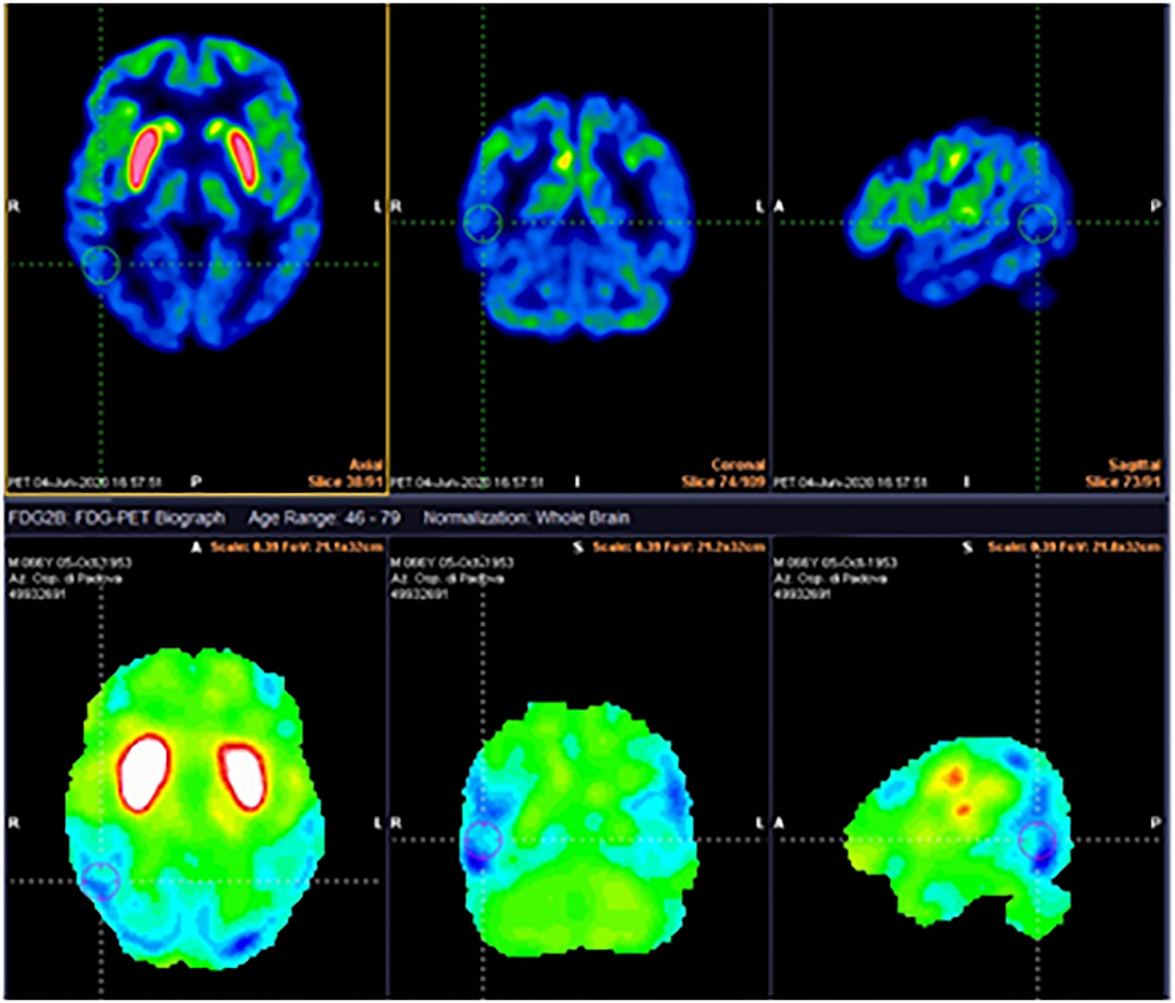
18‐FDG‐PET‐MRI showing marked hypermetabolism in the putamen bilaterally, especially compared to the caudate that appears less enhanced, and hypometabolism in the parieto‐temporal and occipital cortex bilaterally.

Simultaneous FDG‐PET‐MRI (Fig. 1) showed marked hypermetabolism in the putamen bilaterally, especially compared to the caudate that appeared less enhanced, and hypometabolism in the parieto‐temporal and occipital cortex bilaterally. A further testing for antiphospholipid antibodies found slightly elevated aCL‐IgM and moderately elevated β2‐GPI‐IgM; this result was confirmed on two separate occasions three months apart, suggesting a diagnosis of APS‐related chorea. Tetrabenazine, aripriprazol, haloperidol and clonazepam were ineffective. A moderate subjective benefit and a global reduction of the chorea was achieved after starting anticoagulation with warfarin.

### Case 2

A 63‐year‐old female presented with an 8‐year history of relapsing episodes of chorea and dysarthria and recent decline in memory and language. She had a previous diagnosis of antiphospholipid syndrome formulated based on a remote deep venous thrombosis and mitral valve disease requiring surgical replacement, along with positive laboratory criteria. She had a long history of migraine with visual aura. Her renal function was moderately impaired due to membranoproliferative glomerulonephritis. Additionally, she suffered from large B cell lymphoma with metastatic spread to the lung and spleen, treated with R‐COMP chemotherapy. She was born preterm at 7 month and the family reported some difficulties in school due to mild intellectual disability. At age 62, she underwent brain MRI, which revealed multiple degenerative‐atrophic cortical–subcortical areas consistent with ischemic strokes in the occipital, frontal and parietal lobes of the right hemisphere and in the cerebellum bilaterally. She was on warfarin and on multiple pharmacological treatment for the heart and kidney.

On admission, she presented with slight dysarthria, moderate gait and limb ataxia, mild choreic movements of the oromandibular region and of the face and neck (Video 2); a neuropsychological assessment revealed insufficient performance in memory, attention and executive functions.

**Video 2:** Patient 2 Neurological Examination. Video showing choreic movement of the oromandibular region and of the face and neck.

Cerebral spinal fluid (CSF) showed normal biochemical analysis and cytology, but increased levels of total tau protein with normal levels of phosphorylated tau (P‐tau) and beta‐amyloid. Antineuronal and neuronal surface antibodies were normal. Blood tests confirmed the presence of high levels of anti‐cardiolipin antibodies (IgG), anti‐beta2‐glicoprotein‐I (IgG) and a positive LAC. Given disease history characterized by periods of remission of chorea, along with brain imaging pointing to multiple vascular accidents and considering the previous diagnosis of APS, genetic causes of chorea such as Huntington's disease genetic testing was not performed. She underwent a whole‐body PET‐CT that revealed an intense hypermetabolism in both striata (more on the right side) along with hypometabolism in the left parieto‐occipital region. Aripriprazole was started with little benefit on the hyperkinetic movements.

## Literature Review

### Methods

We conducted separate PubMed database searches for English language articles, utilizing the terms “Antiphospholipid syndrome” and “chorea” (166 results), “Antiphospholipid syndrome” and “dyskinesia” (90 results), “APS” and “chorea” (84 results), “ACL antibodies” and “chorea” (22 results), “anti‐β2‐glycoprotein I antibodies” and “chorea” (3 results), “LAC” and “chorea” (3 results). We selected articles published between 1991 and January 2021. Excluding duplicates and articles that were not in English, we obtained 180 articles.

All articles were screened by reviewing the title and abstract to determine if they fell within the scope of this paper. Articles were excluded if no abstract was available, or if they lacked relevance (not related to chorea or antiphospholipid syndrome, outdated information, or redundancy). From the total 180 articles identified, 65 were selected for further review. Clinical cases were reported in 30 articles, for a total of 140 patients affected by antiphospholipid‐related chorea.

Data from these papers were summarized using a standardized data form, including sex, age of onset, type of APS (primary, secondary), subtype of antibody (aCL, beta2GPI, LAC), subtype of chorea (focal‐hemichorea, generalized), structural imaging (CT or MRI), functional imaging (PET CT or PET MRI), therapy, evolution.

We performed additional targeted research regarding functional imaging in antiphospholipid‐related chorea, utilizing the terms “antiphospholipid” and “chorea” and “PET” (8 results), “antiphospholipid” and “chorea” and “functional imaging” (0 results), “antiphospholipid” and “chorea” and “functional magnetic resonance imaging” (0 results), “antiphospholipid” and “chorea” and “positron emission tomography” (6 results) “antiphospholipid” and “chorea” and “single photon emission computed tomography” (2 results), “antiphospholipid” and “chorea” and “SPECT” (2 results). Excluding duplicates of the former research, 4 further articles were added to our library (two of them were additional APL‐related chorea clinical cases).

## Results

Among 142 clinical cases of antiphospholipid‐related chorea found in literature, only 10 had undergone brain metabolic imaging. Striatal hypermetabolism (bilaterally in generalized chorea or contralateral in hemichorea) was found in all cases^6^ who underwent FDG‐PET cerebral imaging (Table 1). Cerebral perfusion single photon emission computed tomography (SPECT) was normal in two cases, while the other two presented with basal ganglia hypoperfusion (Table 2).

**TABLE 1:** Brain FDG PET results in patients with antiphospholipid‐related chorea found in literature

Patient	Reference	Chorea localization	CEREBRAL FDG‐PET results
1	6	Hemichorea (two episodes, one left and one right)	FDG‐PET‐CT: Contralateral striatal hypermetabolism
2	7	Chorea predominantly affecting the right side of face and body	FDG‐PET‐CT: bilateral lentiform and caudate nucleus hypermetabolism (more on the left side)
3	8	Alternating hemichorea	FDG‐PET‐CT: altered striatal metabolism in his left putamen while he was exhibiting right‐sided hemichorea
4	9	Hemichorea (left side) evolving to generalized chorea	FDG‐PET‐MRI: bilateral striatal hypermetabolism
5	10	Hemichorea (right side)	FDG‐PET‐CT: increased bilateral striatal metabolic activity, more on the left side
6	11	Generalized chorea	FDG‐PET‐MRI: increased metabolism in the basal ganglia and motor cortex bilaterally
7	Case report n.1	Generalized chorea	FDG‐PET‐MRI: marked hypermetabolism in the putamen bilaterally and hypometabolism in the parieto‐temporal and occipital cortex bilaterally
8	Case report n.2	Generalized chorea	FDG‐PET‐CT: intense hypermetabolism in both the striata (more on the right side), hypometabolism in the left parieto‐occipital region

**TABLE 2:** Brain SPECT results in patients with antiphospholipid‐related chorea found in literature

Patient	Reference	Chorea localization	Cerebral spect results
1	12	Hemichorea (right side) evolving to generalized chorea	Normal
2	13	Generalized chorea	Hypoperfusion in the basal ganglia and in the medial parts of both temporal lobes
3	14	Generalized chorea	Hypoperfusion of the right basal ganglia. Minor left parietal cortex hypoperfusion and frontal periventricular hypoperfusion.
4	15	Generalized chorea (left side > right side)	Normal

## Discussion

Striatal hypometabolism at FDG‐PET has been documented in both asymptomatic carriers and clinically manifest Huntington's disease as well as in other neurodegenerative choreas like chorea‐acanthocytosis, McLeod syndrome, spinocerebellar ataxia 17) and disease is explained by the progressive synaptic dysfunction and neuronal loss in this region. By contrast, striatal hypermetabolism has been found in several cases of chorea due to potentially reversible causes (Sydenham chorea, antiphospholipid‐related chorea, chorea due to hyperthyroidism)^6^ although the mechanisms leading to the clinical presentation of chorea and metabolic changes in inflammatory/autoimmune diseases are still debated. Peluso et al.^5^ suggested that increased synaptic activity and glucose uptake may be caused by infiltrated lymphocytes and resident microglial cells, delineating a flogistic state, while Ehrlich et al.^16^ hypothesized that striata hypermetabolism could be a sign of compensatory changes which could lead to the eventual resolution of chorea. In two of the clinical cases reported in Table 1 (patients 2 and 4), a follow‐up functional imaging showed that with the resolution of the chorea, striata metabolism returned to normal levels.

Our clinical cases are consistent with the literature findings on FDG‐PET imaging. Particularly in the first case, we showed how cerebral FDG‐PET can have a role not only in better understanding the mechanism of antiphospholipid‐related chorea, but also in the diagnostic work‐up of chorea of unknown etiology. When a patient's clinical presentation is not clearly suggestive of either a neurodegenerative or a reversible cause of chorea, and additional first‐line investigations are normal, FDG‐PET can help distinguishing the underlying etiology, especially in presence of striatal hypermetabolism.

We decided to add the literature data about perfusion SPECT, to evaluate if striatal hypermetabolism in antiphospholipid‐related chorea could be coupled with changes of blood perfusion in the same structures. SPECT data are less numerous and show inconclusive results with either normal scans or basal ganglia hypoperfusion.

It has been suggested that aPL could bind to brain blood vessel endothelium, causing endothelial dysfunction, leading to microthrombosis and inflammation.^17^ While striata hypermetabolism may be result from an inflammatory process, an explanation for basal ganglia hypoperfusion may be local microthrombosis; this may explain the improvement of some patients after starting anticoagulant therapy.

These two possible pathogenetic mechanisms are sustained by the treatments that are currently used for APL‐related chorea, that include symptomatic drugs (neuroleptics, tetrabenazine), but also steroids and other immunosuppressants and anticoagulant/antiaggregant agents. We suggest that the different response to the treatment may be explained by the relevance of either pathogenetic mechanism.

In conclusion, when a patient's clinical presentation is not clearly suggestive of either a neurodegenerative or an autoimmune cause of chorea, and first‐line investigations are normal, FDG‐PET can assist in narrowing the differential diagnosis, particularly in presence of striatal hypermetabolism.

## Author Roles

1. Research project: A. Conception, B. Organization, C. Execution.

2. Manuscript Preparation: A. Writing of final draft, B. Review and Critique.

LL: 1A, 1B, 1C, 2A.

SA: 1A, 1C, 2A.

GB: 2B.

MM: 2B.

MC: 1A, 1B, 2B.

AA: 1A, 2B.

## Disclosures

### Ethical Compliance Statement

The authors confirm that the Ethics board clearance was not required for this work. The subject has provided written video consent. We confirm that we have read the Journal's position on issues involved in ethical publication and affirm that this work is consistent with those guidelines.

### Funding Sources and Conflicts of Interest

No specific funding was received for this work and the authors declare that there are no conflicts of interest relevant to this work.

### Financial Disclosures for the Previous 12 Months

Angelo Antonini has received compensation for consultancy and speaker related activities from UCB, Boehringer Ingelheim, Ever Pharma, General Electric, Britannia, AbbVie, Kyowa Kirin, Zambon, Bial, Theravance Biopharma, Jazz Pharmaceuticals, Roche, Medscape; he receives research support from Bial, Lundbeck, Roche, Angelini Pharmaceuticals, Horizon 2020 ‐ Grant 825785, Horizon2020 Grant 101016902, Ministry of Education University and Research (MIUR) Grant ARS01_01081, Cariparo Foundation, Movement Disorders Society for NMS Scale validation. He serves as consultant for Boehringer–Ingelheim for legal cases on pathological gambling.
